# PD92 Procedures For Disinvestment Of Health Technologies In Latin America

**DOI:** 10.1017/S0266462325102183

**Published:** 2025-12-29

**Authors:** Magdalena Irisarri, Ana Deminco, Javier Pintos, Alejandra Croci, Alejandra Ferrari, Gervasio Cabrera, Ana Pérez Galán, Alicia Alemán

## Abstract

**Introduction:**

The reallocation of resources to efficacious and safe health technologies (HT) using disinvestment strategies to identify technologies considered to have low health value is relevant to ensure efficient use of resources. The objective of this work was to identify HT disinvestment strategies in Latin American countries and to describe the procedures used.

**Methods:**

Participants in the 15th meeting of the Health Technology Assessment Network of the Americas (RedETSA), held in November 2024, were interviewed. Representatives of agencies from 10 Latin American countries (Argentina, Chile, Colombia, Cuba, Dominican Republic, Ecuador, El Salvador, Mexico, Peru, Uruguay) answered the following open questions: (i) Is disinvestment performed in your country?; (ii) Do you have established procedures for disinvestment?; (iii) How do you select HT to be disinvested?; and (iv) What type of HT are remove from the coverage of benefits?

**Results:**

Argentina and Colombia have established procedures and have used them to implement HT disinvestment. Mexico also has procedures, but they have been used rarely. El Salvador does not have explicit procedures, but mechanisms for disinvestment have been used following requests from different stakeholders. The rest of the countries do not have established procedures but have excluded HT due to obsolescence or occurrence of adverse events that were previously unknown. Health technology assessment has been used to remove coverage for obsolete technologies and indications not previously assessed. Most technologies disinvested using health technology assessment were high-priced oncological medications.

**Conclusions:**

In Latin America, very few countries have explicit procedures to identify and disinvest inefficient HT. Since the reallocation of resources is a well-known tool to improve the efficiency of health systems, it is necessary to establish well defined procedures to identify potential technologies for disinvestment and to remove their funding in the different health systems.

